# From the Horse’s Perspective: Investigating Attachment Behaviour and the Effect of Training Method on Fear Reactions and Ease of Handling—A Pilot Study

**DOI:** 10.3390/ani11020457

**Published:** 2021-02-09

**Authors:** Elke Hartmann, Therese Rehn, Janne Winther Christensen, Per Peetz Nielsen, Paul McGreevy

**Affiliations:** 1 Department of Animal Environment and Health, Swedish University of Agricultural Sciences, 75007 Uppsala, Sweden; therese.rehn@slu.se; 2 Department of Animal Science—ANIS Welfare, Aarhus University, 8830 Tjele, Denmark; jwc@anis.au.dk; 3 Department of Agriculture and Food, RISE Research Institute of Sweden, 22370 Lund, Sweden; per.peetz.nielsen@ri.se; 4 Sydney School of Veterinary Science, Faculty of Science, University of Sydney, Sydney, NSW 2006, Australia; paul.mcgreevy@sydney.edu.au

**Keywords:** equine, handling, attachment, bond, behaviour, welfare, safety

## Abstract

**Simple Summary:**

Optimising horse–human relationships can promote positive experiences and advance the welfare and safety of both dyad members. Attachment and bonding are key components in such relationships, and horses are good candidate subjects for studying bonding processes due to their social nature, artificial selection for trainability and their dependence on human care in a domestic context. However, the factors that contribute to successful relationships remain unclear. This preliminary study on 12 horses investigated whether horses develop an attachment bond with their trainer after a short period of frequent interactions. The study also aimed to explore how the type of training method (negative reinforcement and two types of combined reinforcement) may affect the horse–human relationship and how this manifests as ease of handling in a novel environment. The horses showed reduced reactions in both the fear test (encountering novel objects with the trainer and a stranger present while moving freely) and handling test (encountering novel objects while being led by the trainer versus a stranger) after training compared to before training. However, we could not provide conclusive evidence that horse–human relationships established during training constitute an attachment. Suggestions for future studies are provided.

**Abstract:**

The study investigated equine responses to novelty and handling, aiming to reveal whether horse–human relationships reflect criteria of an attachment bond. Twelve adult Standardbreds were subjected to a fear-eliciting test (novel objects presented close to two humans) and a handling test (being led passing novel objects) to study attachment-related behaviours and ease of handling. The tests were performed both before (pre-test) and after (post-test) horses had been trained by the same female handler (10 sessions of 15 min). Horses were assigned to three groups of four, each of which underwent different operant conditioning protocols: negative reinforcement (NR; pressure, release of lead, and whip tap signals) or combined NR with either positive reinforcement using food (PRf) or wither scratching (PRs). Results showed that neither familiarity of the person nor training method had a significant impact on the horses’ behavioural responses in the post-tests. However, horses showed decreased heart rates between pre- and post-tests, which may indicate habituation, an effect of training per se, or that the presence of the familiar trainer served to calm the horses during the challenging situations. There were large individual variations among the horses’ responses and further studies are needed to increase our understanding of horse–human relationships.

## 1. Introduction

Horses are flight animals by nature and usually react with avoidance behaviour in fear-eliciting situations. Therefore, reducing fearfulness so that the horse is responsive to human signalling is highly desirable in most training systems. It also has relevance for welfare and safety, because most behavioural fear reactions increase the risk of injury for both dyad members [1,2]. Moreover, as suggested by much anecdotal discourse that speaks of trust, the establishment of a reciprocal bond between handler and horse may further reduce fear and its undesirable manifestations in both parties [3].

It has been proposed that training a horse to be under stimulus control (i.e., it responds readily and reliably to light signals from a rider or handler) may over-shadow inherent fear responses. This means that the horse has a stronger motivation to respond to anthropogenic signals than to exogenous cues from the environment [4,5]. However, scientific evidence of the effects of the handler’s familiarity on equine fear reactions during handling is contradictory. In a recent study, an unknown handler was as effective (no difference in behavioural and physiological stress responses) as the horse’s familiar handler during stressful handling procedures (walking over tarpaulin and beneath plastic streamers). This indicates that the horse’s performance was unaffected by the chronicity of the relationship between horse and human [5]. In contrast, being handled by a known handler (familiarity having been established during eight consecutive training sessions prior to testing) increased compliance in previously unhandled test horses, i.e., there were fewer behaviours indicative of fear while passing between novel objects than when being handled by a stranger [4]. Clearly, understanding how horses experience the relationship with their owner, main caregiver or other humans of varying familiarity merits further investigation because it can help to clarify the concepts of mutual trust and affection and in moderate human expectations in different training situations.

A relationship is described as an association between two individuals over time [6], which can have the characteristics of an affectional bond, i.e., a long-lasting tie in which the partner is important as a unique individual and is not easily interchangeable with someone else [7]. Attachment is defined as an affectional bond with the addition of security and comfort gained from the relationship [7]. In human psychology, attachment of a child to its main caregiver (attachment figure) has been extensively investigated [8,9]. Behaviours indicative of attachment have been described by considering the balance between two motivational systems, i.e., protection from threat (proximity- and comfort-seeking from the attachment figure, their ‘safe haven’) and the willingness to explore the environment (ability to move away from the attachment figure once comfort is gained, the ‘secure base effect’ [10]). In contrast to an attachment bond, which can be described by a specific behavioural strategy directed only to a few individuals, random attachment behaviours can be expressed to many people or objects in a variety of contexts [8].

The attachment bonds of animals towards humans have been explored mainly in companion dogs, and cats, and recently also wolves and horses [11,12,13,14,15], using established methods derived from human psychology [9,16]. Studies in domestic dogs and cats suggest that these species can manifest attachment bonds toward their caregiver [11,12,17,18]. This is based on results from so-called Strange Situation Procedure (SSP) tests, where animals are exposed to challenging events (separation from and reunion with a familiar person and meeting a stranger in a novel environment). The animal’s proximity-seeking to the caregiver or the stranger in times of distress is recorded and metrics include, e.g., how often the animal engages in play or explores a novel environment when the caregiver is present versus absent [12,17].

Horses share a long history with humans and merit studies of their perspectives of relationships. This can be investigated by exploring horses’ responses to their main caregivers, even though horses do not share the same domestic quarters with humans as most companion dogs and cats do. Nevertheless, many caregivers may consider their horses to be part of the family and would relate to them as they would to a family member, taking care of them because of this affective connection [19]. The caregiver may express affection, e.g., by scratching and grooming the horse, whereby touch is posited to be an important ingredient for forming attachment-like bonds [20]. Whether the horse reciprocates those grooming attempts due to affection for its caregiver or simply because of innate tendencies to reciprocate grooming that may protract a reinforcing intervention, remains open for investigation. Moreover, like dogs, most domestic horses are social animals that largely rely on human caregiving for survival. Thus, the formation of a social relationship based on attachment seems plausible [3]. There are fragments of evidence that may indicate such bonds. For example, one study showed that human presence could reduce agitation in horses in that they were emphatically more willing to approach a novel object while being led by a handler than when alone [21]. The authors did not specify the familiarity of the handler to the test horses. Therefore, whether the results reflect a secure base effect or simply that horse handling in general facilitates habituation remains unclear. A recent study [13] revealed that horses sought human proximity and heart rates were reduced upon reunion with the owner, i.e., their main caregiver and a stranger, suggesting that horses regard both the caregiver and the stranger as a safe-haven.

Previous experiences with humans can affect horses’ reactions towards them [22,23] and potentially mediate the establishment and quality of bonds between horse and human. Positive affect may be fostered via appropriate husbandry protocols but also by the use of appropriate training practices. In dog training, providing food, play or physical contact as rewards (positive reinforcement, PR) has become standard and has largely replaced dominant and coercive styles of training [24,25]. In horses, using PR during training has increased in popularity although negative reinforcement (removal of an unpleasant stimulus such as leg or bit pressure to reward desired behaviour, NR) cannot be completely abandoned if horses are to carry a rider or pull a carriage where communication is merely based on pressure cues rather than other modalities such as voice commands. Sankey et al. [26,27] attempted to explore the effect of PR training on the horse–human relationship and compare it with aversive events. Horses receiving food rewards during training spent more time close to the trainer than to an unfamiliar experimenter and approached the trainer faster than horses trained without food or solely trained with NR. Compared with vigorously scratching at the withers, food as a primary reinforcer accelerated learning and promoted bonding as measured by the latency to approach a human and the time spent near her [28]. Nevertheless, empirical evidence suggests that, at times, tactile contact seems important in affiliative interactions between horses, which may also apply to human-horse interactions, as shown in declined heart rates and more relaxed type of behaviours expressed in horses after prolonged grooming by humans [29,30]. However, whether horses perceive human touch as social bonding remains debatable [31]. For example, in a training context, scratching the withers vigorously three times [28] or for up to one minute [32] may not be perceived by the horse as sufficiently positive or rewarding to enhance learning or facilitate bonding. One should also consider that there are individual differences in how tactile contact is experienced [33] or, equally, how food is more reinforcing for some individuals than others [31,34]. Since adding food rewards may not appeal to all horse caregivers and trainers, the effect of using NR alone in a training context on the horse–human bond and training outcomes warrants further inquiry.

The aim of the current pilot study was three-fold. Firstly, we wanted to explore whether horses would show attachment-like behaviours to their trainer as a consequence of repeated interactions (10 training sessions, each lasting 15 min). This was assessed while exposing horses to a stressful test situation involving social isolation from conspecifics in an arena with novel objects while both the trainer and a stranger were present. We hypothesised that if horses established an affectional bond with the trainer, they would show attachment-related behaviour towards her preferentially, i.e., horses would spend more time in proximity to the trainer than the stranger (safe haven effect) and investigate the novel objects closer to her than those closer to the stranger (secure base effect). Second, we aimed to assess the horses’ reactions while being led by the trainer as compared to a stranger through a parkour consisting of five stations with novel objects and a sudden noise. We hypothesised that if horses established a bond with their trainer, then this would translate back to improved compliance during handling (fewer resistance behaviours). Third, we aimed to explore the effect of training method on the horse-trainer relationship and ease of handling. Thus, we hypothesised that adding food rewards (PRf) to NR training has the greatest potential to ease handling as it increases the horse’s motivation to participate with and focus on the trainer. Moreover, we predicted that combining NR with the provision of food (PRf) or scratching the withers (PRs) versus using NR alone to reward correct responses to lead and whip pressure cues may lead to more positive associations with the trainer, which may facilitate the formation of an affectional bond.

## 2. Materials and Methods

The experiment was conducted between March and May 2018. Experimental procedures conformed to the guidelines for the ethical treatment of animals in applied animal behaviour research (http://www.applied-ethology.org/ethical_guidelines.html; March 2018) and were approved by Uppsala’s local Animal Ethics Committee under the protocol A 14-2016.

### 2.1. Horses, Facilities, and Handlers

Twelve Standardbreds (six mares, six geldings) between five and 13 years (Mean ± SD: 9.3 ± 2.3) were used in this study. All horses were housed at Wången, the Swedish National Centre for the education and development of harness racing and Icelandic horse riding. They were school horses and were regularly driven and ridden applying the same training approach (i.e., mainly use of NR whereas PR was not part of the general training regime) by different students who were also responsible for their routine care. The horses were stabled in individual box stalls (3 × 3 m) and were turned-out in pairs or groups during daytime for 4–6 h. They received individually adapted feeding rations of 10–12 kg forage (haylage) and concentrate feed including mineral and vitamin supplements (Krafft, Malmö, Sweden) divided over four feeding occasions per day. Water was available ad libitum. All horses were held at Wången for at least two years prior to this study. Horses with behavioural abnormalities or handling problems were excluded from the study.

The experimental tests were conducted in the school’s indoor arena (60 × 30 m) which was visually separated from an adjacent hall, containing three box stalls (3 × 3 m) and an open, concrete area of around 15 × 20 m. In this hall, the boxes were used for preparing horses (fitting heart rate [HR] equipment) prior to testing in the indoor arena, and the concrete area was used for the training sessions. The boxes were also used for testing horses’ reactions to wither scratching by a human and their motivation to consume pelleted feed (200 g) from a bucket, which was part of their regular diet. Both the indoor arena and the adjacent hall were familiar to the horses. None of the horses had to be habituated to wearing an elastic girth with HR equipment as HR was regularly monitored during physical conditioning on Wången’s racetrack. Horses wore a regular halter during all experimental tests as well as during the training sessions.

All handlers involved in this study were female and experienced with horses. They always wore safety clothing (helmet, gloves, and protective shoes). The three handlers (two unfamiliar handlers and one trainer unknown to the horses prior to the experimental period) participating in the experimental tests wore the same clothes during pre-, and post testing and during the training sessions. Additionally, two students were responsible for preparing the horses for the tests and for leading them into the indoor arena and back, and another person was responsible for filming.

### 2.2. Experimental Setup

**Figure 1:**
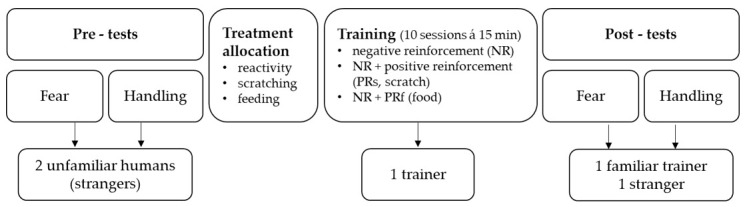
Overview of the experimental set-up (order from left to right) consisting of the three phases: (1) pre-tests, (2) training, and (3) post-tests.

The experiment consisted of three phases, i.e., two pre-tests (measuring horses’ fear reactions and ease of handling), a training phase, and two post-tests (repeating the fear and handling test). In the first phase, horses’ reactions to novel objects were investigated in the presence of two unfamiliar handlers neither of whom had any prior contact with the horses (fear pre-test). Subsequently, their reactions while being led by the same two handlers were measured as they passed other novel objects (handling pre-test). This was followed by a training phase of 10 days, consisting of 10 standardised training sessions (duration 15 min). During the sessions, correct responses to light pressure applied via the lead-rope and from whip-taps on certain body parts (shoulder, ribcage, hock, croup) were rewarded by using solely NR, using NR in combination with PR in form of food (PRf), and using NR in combination with PR in form of scratching of the withers (PRs); see further description below and Figure 1 and Table 1. Thus, horses were assigned to groups of four horses each, according to one of the three training methods. The same person, who had also been one of the unfamiliar handlers in the pre-tests (hereafter called ‘trainer’), trained all horses. In the third phase, the fear and handling tests were repeated (fear and handling post-test) with another unfamiliar handler and the familiar trainer directly after the training phase. Between pre-tests and post-tests and within consecutive handling tests, the novel objects differed in layout and number to avoid habituation due to repeated exposure.

#### 2.2.1. Fear Test

**Figure 2:**
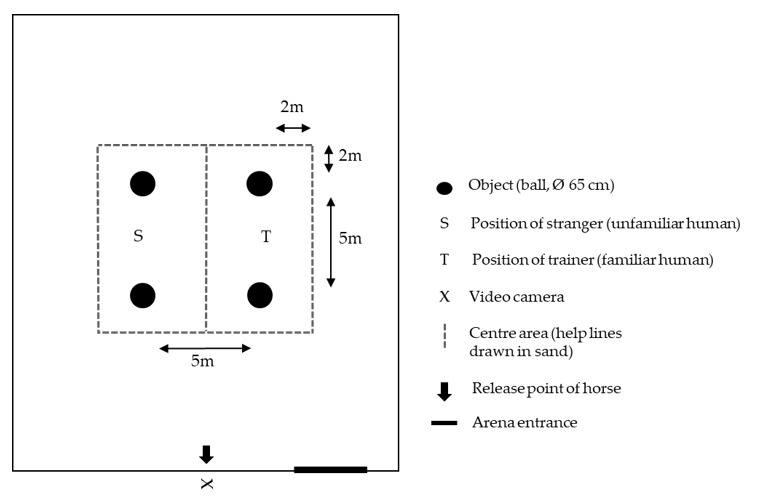
Experimental area (not in scale) and set-up during the fear pre- and post-test. Outer black lines indicate the walls of the indoor arena (60 × 30 m). Dashed lines (drawn in the sand of the indoor arena) indicate the centre area near the stranger (S) versus the trainer (T) used for behavioural analysis from video recordings.

In the fear pre-test, four identical pink rubber balls (65 cm diameter) were placed in the centre of the indoor arena in a square, 5 m apart (Figure 2). The two unfamiliar handlers (strangers) stood between the balls on each side whereby the position of handlers was pseudo-randomised between left and right side for each horse, and balanced across the pre- and post-tests. The handlers were asked to remain motionless and passive, i.e., avoiding physical or vocal interaction with the test horse even if it approached and initiated physical contact.

The test started with the horse being led by a student from the box stall to the indoor arena where it was released (Figure 2). It was allowed to locomote freely and had the opportunity to approach and investigate the objects and/or handlers during 10 min. After that period, the horse was caught and returned to its box stall and the next horse was taken to the indoor arena for testing.

The set-up for the fear post-test was the same as for the fear pre-test. However, the four rubber balls were wrapped into blue plastic bags and one of the handlers, who initially had been unfamiliar to the horses, became the familiar trainer.

#### 2.2.2. Handling Test

**Figure 3:**
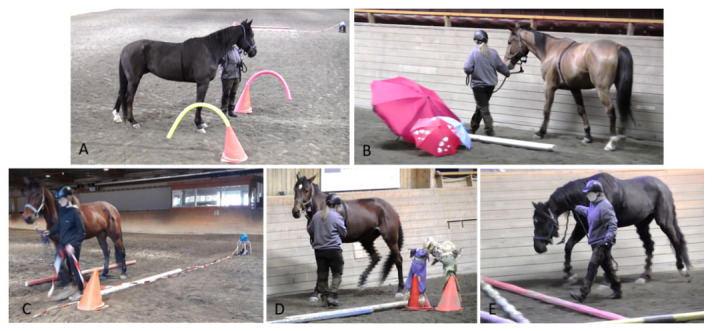
Handling test, showing different horses (fitted with a regular halter including HR equipment) passing, always in a counter-clockwise direction, five stations with novel objects while being led by a handler. (**A**) The horse was cued to stand still for 5 s between two traffic cones bearing curved foam projections. (**B**) The horse was led through a 1.5 m wide and 2 m long corridor between the arena wall and objects. (**C**) The horse had to pass a plastic bag placed on a chair and was then stopped at a traffic cone 3 m behind the bag. The handler took a rope attached to the bag and pulled it once so the bag, filled with plastic bottles and metal cans made a noise as it fell a height of 40 cm from the chair to the ground. (**D**) The horse had to pass cones bearing objects and was cued to halt at the end of the 5 × 2 m corridor. Then, the handler turned around, facing the horse and applied lead pressure to cue the horse to step backwards. Every backward step was rewarded by releasing pressure. The task stopped when the horse’s hindquarters were level with the objects. From that position, the horse was again led forward, approaching the last station. (**E**) The horse was cued to step over rails on the ground. (Photo courtesy Elke Hartmann, Veera Marianna Valtanen).

In the handling test (Figure 3), horses had to pass, always in the same order, five different stations containing novel objects (visual and auditory stimuli) while being led separately through the parkour by two handlers in two successive trials (5–10 min break between trials). The order of handlers was determined pseudo-randomly for each horse but balanced across the pre- and post-test. The horse was always led from the left side. The lead rope hung loose during leading, but tension was applied when the horse was cued to move forward, stop, or back up. Tension was released immediately upon correct response or was maintained until the horse offered the desired behaviour. The horse was allowed to observe the objects but not to move away from the direction of travel indicated by the handler.

The set-up for the handling test was the same for the pre-test and post-test, i.e., passing novel objects in the same order and always in the counter-clockwise direction.

#### 2.2.3. Allocation to Training Groups

The horses were assigned to one of the three training groups (NR, NR + PRf, NR + PRs) based on their behavioural reactions to wither scratching, their motivation to consume pelleted food, as well as their reactivity (as expressed in mean HR during the fear pre-test). This was done to balance the number of horses among training groups based on how they perceived wither scratching, their fearfulness, and to ensure that all horses were consuming the food provided since this was used during NR + PRf training.

Before the start of the wither scratching test, the horse was allowed to roam freely for 3 min in the box to settle down prior to being tied up. The horse wore a halter and was loosely tied to the box wall so it was able to express behaviours such as head-lowering (as far as the ground), reciprocal grooming, or head-turning. An unfamiliar handler positioned herself on the horse’s left side, parallel to its withers while facing its head. She firmly massaged the area around the withers for 10, 20 and 30 s in a random order with 30 s pauses between applications. A second person, standing at the door of the box, scored the horse’s behaviour as positive (e.g., sniffing or grooming handler or leaning into her, upper lip movement, relaxation of lower lip), neutral (e.g., indifferent response with no behavioural changes) or negative (e.g., raised head, foot stamping, bite and kick attempts, tense mouth). A companion horse was placed in the neighbouring box so that none of the horses were socially isolated during testing. Since the test horse was tied to the wall opposite to the adjacent box stall, no physical contact between horses through the bars was possible during testing.

Subsequent to the wither scratching test, the horses’ latency to consume 200 g pelleted food (Krafft, Malmö, Sweden) was recorded. A bucket containing the pellets was placed in the entrance of the test box on the floor. The timing (in s) began the moment the horse lowered its head into the bucket and started feeding, and ended when the food was finished or the horse showed no interest in ingesting the remaining food by stepping away from the bucket.

#### 2.2.4. Training Methods

Six horses, two from each treatment group, were trained over a 20-day period such that each of the 12 horses had one day off between training sessions. Each horse received 10 training sessions corresponding to 10 days of training whereby each session lasted for approximately 15 min. The training took place during late afternoon/evening between 17:00–20:00, after horses had returned from the paddocks and been fed, and after school activities that included regular racetrack training of the horses by Wången’s students. The trainer had no contact with the horses outside the training sessions.

For a training session, horses were equipped with a halter and taken directly from the home box to the hall adjacent to the indoor arena where all training took place. During a training session, no other horse was in sight by the horse being trained. All horses were treated in the same manner, i.e., no additional stroking, food rewards or talking to the horse other than specified as part of the training method.

The training included tasks that many horses experience during basic routine ground-work where they had to respond to pressure cues from the lead rope (tasks 1–4, 7, 10) and to whip-tap signals (tasks 5–6, 8–9; Table 1). A new exercise was introduced at each session, whereby the training started with a short repetition of the previously learned task.

Horses were trained from both sides, starting on the left side and moving to the right side when the horse responded correctly to a light signal. The signal given via the lead-rope or whip-tap always started with light pressure that was gradually increased in frequency until correct response. Pressure was released immediately to reward the desired behaviour. Furthermore, the word ‘good’ was used to signal the correct response and, according to the training method, horses were hand-fed a small amount of pellets or were scratched firmly at the withers for 5–10 s after every correct response. Learning criteria included an immediate response to light lead pressure or to gentle whip-tapping of maximum five repetitive taps. The trainer herself subjectively evaluated the horse’s responses to be able to complete the task from the other side. Horses received short breaks during a session by walking them around for a few steps before returning to training the task. All 12 horses underwent the same exercises in the same order, as presented in Table 1 (for more detailed description of the training approach, see [35]).

### 2.3. Recordings and Data Analyses

All fear and handling tests were video-recorded (Canon LEGRIA HF R78 Canon USA, Inc., Melville, NY, USA) for analysis of behaviour (see Table 2) using Mangold Interact Professional software (Version 18.0.2.13, 2017, Mangold International GmbH, Arnstorf, Germany).

HR was recorded with Polar Equine CS600X (Polar Electro Oy, Kempele, Finland). Electrodes, transmitter, and receiver were attached to an elastic girth. To improve conduction between the two electrodes on the girth and the skin of the horse, water and electrically conductive gel (Cefar blue gel) were applied. Data were downloaded to the software Polar ProTrainer 5, Equine edition (Polar Electro OY, Kempele, Finland). Artefacts in HR were corrected using the error correction options of the Polar software. Although recording of HR started at least 5 min prior to all testing, only recordings during the actual tests were used for analysis. For each horse, mean HR and maximum HR were calculated for the 10 min fear pre- and post-tests. For the handling pre- and post-tests, only maximum HR was determined because the duration of passing all stations in the parkour varied for each horse.

Data were analysed in Minitab (version 18.1, 2017; LLC., State College, Pennsylvania, USA) and were tested for normality using the Anderson–Darling test. Data that met the assumptions for variance homogeneity, i.e., HR (bpm) were analysed for treatment effects (training method) using one-way ANOVA, and comparisons between pre- versus post-tests and between trainer and stranger were analysed with paired sample t-tests. Behaviour data were analysed using the Mood’s median test when comparisons were made between treatments. Pairwise comparisons within horse during the fear post-test were made to investigate if horses preferred to seek proximity to and investigate the object close to a particular person (stranger versus trainer). For this, Wilcoxon signed rank tests were applied. Moreover, Spearman correlation tests were used to investigate possible links between HR and behavioural responses of the horses during the fear and handling test. Since our study was explorative in nature, we did not correct for multiple comparisons. The significance level was set at *p* < 0.05.

## 3. Results

In the fear pre-tests, there were no differences concerning location of the horses and distance to the stranger and (unfamiliar) trainer, as expressed in the variable ‘location near person’. In addition, horses investigated the objects regardless of which person was closest. In the handling pre-tests, there were no differences in time passing the parkour according to handler (stranger and [unfamiliar] trainer), nor in the frequency and duration of resistance behaviours.

Of the 12 horses, only four horses investigated the persons, of which one horse did so towards both humans in the fear pre-test, but this horse only investigated the familiar trainer in the post-test. The other three horses investigated only one of the persons each, i.e., two horses investigated the (unfamiliar) trainer in the pre-test and one horse investigated the stranger in the post-test. Hence, no further analyses were performed regarding investigating person.

### 3.1. Effect of Training on Fear Reactions and Ease of Handling

The effect of training, without considering training method, showed that the horses were less aroused as reflected in significantly lower HR in the fear- and handling post-tests than in the pre-tests (Fear test: *p* = 0.03, t = 2.5 [mean HR]; *p* = 0.03, t = 2.6 [max HR]; Handling test with S: *p* = 0.01, t = 3.0 [max HR]; Handling test with T: *p* = 0.02, t = 2.7 [max HR]; see Table 3).

In the fear test, horses visited the stranger’s area less often in the post-test than in the pre-test (*p* = 0.02, W = 8.0). There were no differences in the amount of time spent investigating the object between pre- and post-tests.

In the fear post-tests, horses did not spend more time in proximity to the trainer than to the stranger, and they performed equally well in the handling test regardless of handler familiarity.

### 3.2. Effect of Training Method on Fear Reactions and Ease of Handling

In the fear post-test, there were no differences in the behaviour nor in the HR of the horses related to treatment, i.e., training method (Table 4). In the handling post-test, horses trained with NR + PRs took longer to pass the parkour when handled by a stranger (*p* = 0.02, χ^2^ = 8.00; see Table 4). No other differences were found with regard to training methods.

### 3.3. Correlations between Heart Rate and Investigative Behaviour

In the fear post-test, a negative correlation between mean and max HR and investigating the object close to the trainer was found (Spearman’s Rho = −0.7, *p* = 0.02 [mean HR] and Rho = −0.6, *p* = 0.04 [max HR]). During the fear pre-tests, we found no correlations between HR and investigating the object close to the stranger or trainer. There were no correlations between max HR and behaviour (time passing the parkour and duration/frequency of resistance behaviour) expressed with the trainer or the stranger in any of the handling tests.

## 4. Discussion

The strength and quality of the horse–human relationship have rarely been evaluated in combination with the effects of the type of previous interactions on the quality of the relationship. Thus, we wanted to test whether horses form a relationship with their trainer that shares characteristics of an attachment bond, as expressed in attachment-related behaviours towards her. Moreover, we aimed to investigate how training method influenced the horse’s perception of the trainer as compared to a stranger, and how this translates back into ease of handling in a novel, potentially fearful situation.

The horses in our pilot study did not differentiate between their familiar trainer and strangers for any of the measured variables. Even though we observed attachment-related behaviours, as expressed in approaching humans (resembling a safe haven effect) and investigating the novel objects in their proximity (resembling a secure base) in a few of the horses in the fear post-test, these behaviours were already shown during the pre-test. However, average and maximum heart rates were negatively correlated with investigating the object closest to the familiar trainer in the fear post-test. We found no evidence for an effect of handler familiarity nor training method on ease of handling despite horses that had been trained with combined negative and positive reinforcement (scratching) taking longest to pass the parkour when handled by a stranger. Notably, horses’ heart rates were significantly lower in both the fear and the handling post-tests than in the pre-tests. It is unclear whether this may be an effect of habituation to the test procedures and/or a consequence of training per se.

Our findings with regard to evaluating the horse–human relationship are in alignment with a recent study [13] that reported attachment-related behaviours of horses towards both their owner, i.e., their main caregiver, and a stranger. In that study, horses were exposed to a modified SSP that included separation and reunion from the caregiver and a stranger in a partially novel environment (fenced-off indoor arena). Horses were agitated when the human had left, as shown by spending more time close to the arena entrance and by increased heart rates when compared to the reunion phase [13]. After humans had returned to the test arena, horses sought human proximity and their heart rates decreased significantly. These variables resemble two of the three criteria of an attachment bond [10], namely separation-related distress and safe haven. So, in line with our results, what are the implications of these findings for the prospect of labelling particular horse–human relationships as affectional bonds if they share only some aspects of the traditional attachment theory?

According to Bowlby [8], random attachment behaviours can be shown towards many people or objects in a variety of contexts, which differs to an attachment bond that classically should be a specific behavioural strategy directed to only a few individuals. Thus, applying the term ‘attachment’ rigorously to certain horse–human relationships may not be appropriate as long as horses express attachment behaviours randomly towards various humans, or, as Payne et al. [36] formulated it, horses may show variable alignment with attachment theory. That said, if we are to apply attachment theory for a comparative approach to explain horse–human interactions, then our findings support the view that horses seem to perceive any human as significant players in their environment who may or may not resemble an attachment figure. Horses in our study approached and investigated the novel objects in close proximity to both humans in the fear pre- and post-test, implying that horses were not reliant on the quality of the bond established which also Ijichi et al. [5] have concluded based on their study results. Notably, no other novel objects were presented further away from the humans in the current study. Therefore, we suggest addressing this in future tests that give horses the choice to move further away from the human to explore the environment in order to provide solid evidence of a secure base effect.

Interestingly, attachment-related behaviours to the trainer in the fear post-test were observed only in a few of the 12 horses (n = 5 investigating object close to the trainer, n = 7 located near the trainer). Beside the possibility of various underlying attachment styles, the variation in investigative behaviour among horses may have been caused by individual differences in temperament such as the level of fearfulness or anxiousness [37,38], inquisitiveness [38,39], reactivity to humans [22,40], and previous experiences with humans [41,42]. This is supported by the observation that the lower the horses’ heart rates were, the more they investigated the object close to the familiar trainer. What kind of relationship this may reflect between the horses and their trainer remains debatable because only a few horses accounted for this correlation (five horses investigated the object close to the trainer). Alternatively, because there was no difference in investigative behaviours between the fear pre-test and post-test, this may represent object recognition and generalisation [43,44]. The horses may have habituated to the plastic balls in the pre-test and, therefore, became less disturbed by the presentation of the same shaped object (albeit presented in a different colour) during the post-test. Decreased heart rates in the post-test further support the prospect that horses may have habituated to the experimental set-up due to prior exposure in the pre-test. To activate the behavioural attachment system, a reasonably strong stressor is needed [9]. Thus, if the post-tests were relatively less stress-activating, perhaps they failed to measure attachment at all. Similarly, it is possible that our experimental set-up, i.e., separating horses from conspecifics and releasing them in an indoor arena with novel objects and two passively standing humans, was an insufficient stressor to fully activate the attachment system among multiple horses. Even though social separation from conspecifics constitutes a challenge for many horses [45,46], the adult school horses used in our study may well have experienced repeated separation from companions previously during training, veterinary care or transport. Likewise, wariness and consequent avoidance of strangers may be generally low in horses that have been frequently handled and trained by many humans, especially if repeated interactions are perceived by the horse as positive [27,42]. Merkies et al. [47] found that therapy horses clearly preferred the presence of any human as compared to being left alone in a round-pen. As said before, the horses used in our study were school horses and thus used to being handled by many different humans which may affect the likelihood of establishing a relationship as compared to, e.g., previously unhandled horses [4].

The current finding that a few horses had lower heart rates when investigating the object closest to their trainer raises the question of whether they regarded the trainer as more experienced or reliable than the stranger, based on familiarity and previous calm and competent handling, or whether other human attributes affected their responses. Since humans in our test stood passively and were not allowed to talk to the horses, interact with or touch them, we assume that body posture did not influence the horses’ responses [48,49]. However, beside the possibility that horses perceived passive humans as objects or that they could not recognize the familiar trainer from the stranger at least from a distance, one cannot rule out the possibility that an array of other human behaviours and attributes modulated the horses’ reactions. These could include unintentional cueing [50], emotional expressions [51], emotional intelligence [3], or attitudes towards horses [52], and other putative influences such as conditioned safety signals [53]. We propose to address these aspects in future research to increase our understanding of how horses perceive humans. In dogs, Rehn and Keeling [18] suggested including human behaviour in the analysis of the dog–human relationship because it is likely to affect the dog’s behaviour.

Training, in the form of 10 repeated 15-min sessions every other day with the same person had no effect on the horses’ responses in the fear post-test. One of the main purposes of the training was to allow horses to form a relationship with the trainer and increase the chances of an attachment bond developing. Consistent and appropriate interactions aligned with learning theory may have created positive affect, thereby encouraging approach behaviour of horses to the trainer but also to strangers [3]. Even though approach behaviour was generally low, it was shown irrespective of familiarity with the human, so our data may support this idea. Heart rate was lower in horses approaching the familiar trainer, which may be an indirect measure of positive affect. Additionally, heart rate was lower for horses in the post-test, which may indicate that the mere presence of a familiar person had a calming effect on the horses, but this did not manifest in increased investigation of the objects nor more proximity-seeking.

The current length of training interactions (10 sessions of 15 min), we argue, may have been sufficient to establish a relationship with the trainer. In dogs, Gácsi et al. [54] showed that adult shelter dogs expressed attachment-related behaviour to their familiar handler, evaluated in a modified SSP test, reasonably quickly, after only three 10-min handling sessions. In horses, Marsbøll and Christensen [4] exposed previously unhandled horses to a maximum of eight, 20-min training sessions. The authors found an effect of handler familiarity, i.e., shorter duration of resistance behaviours (standing still or moving away from the intended direction) with the known, female trainer as compared to an unknown male handler while passing through a corridor of novel objects. The latter result is contrary to our findings because resistance behaviours and time passing the novel parkour were not affected by handler familiarity as in the study by Ijichi et al. [5]. Ijichi et al. [5] concluded that the quality of the bond with a familiar human (i.e., the horse’s main caregiver) did not influence compliance during handling. So, effective and thereby safe handling is achievable even in situations where bonding is not an option, e.g., during veterinary procedures or therapeutic sessions were horses meet humans and patients with whom they have not previously interacted. This may be contrary to what has been found in dogs. Dogs differed in their compliance with operant cues from familiar versus unfamiliar people, and the owner was markedly more effective than strangers in calming dogs during fearful situations [55] and decision-making [56]. However, in less challenging experimental environments, social familiarity seems less important for dogs [57]. If horses in the current study experienced the fear and handling post-tests as less challenging, they may have generalized the generic qualities of the trainer and stranger. Nevertheless, horses with lower heart rates in the fear post-test seemed to choose to investigate the object close to the trainer (or the heart rate was reduced as an effect of the closeness of the trainer). Despite similarities in sociality and trainability of horses and dogs, there are several important differences between these species and their responses to training [58]. Even though both species are social, dogs are mainly predators while horses are prey animals, suggesting differences in the responses to a sudden stressor (in that a flight response is generally more likely among horses). Additionally, horses do not normally live in such close contact with an owner/trainer as most dogs do, which may affect the relationship but even more so, their ability to read human gestures and intentions, which is important in order to establish attachment bonds [59]. Further studies may explore the optimal balance of arousal and affective state for the tests imposed [60] and eventually decipher the role of horse–human attachment in training outcomes [61].

Another purpose of the current training was to assess the effect of training method on the quality of the relationship and ease of handling as measured in time passing the parkour and the frequency and duration of resistance behaviours. We tested three different approaches, namely training horses exercises in hand by applying negative reinforcement only, and by using combined negative and positive reinforcement in form of food rewards or scratching the withers. Results revealed no differences in behavioural and cardiac responses with respect to training method when tested with the familiar person. However, horses reinforced with both the release of lead tension and whip-tap pressure and an added scratch on the withers (NR + PRs) took longest to be led through the parkour by the unfamiliar handler. It was hypothesised that human-induced grooming may facilitate bonding due its characteristic of an affiliative action [3] and its beneficial effects on heart rate [30] as well as behavioural responses [32,62]. Still, this training approach may benefit cooperation in a handling context with a handler sharing a longer history with the horse than the limited amount of sessions applied in the current study. Whether this would be a consequence of affect or simply an innate response to being groomed remains open for investigation. Interestingly, when horses are given the choice, they prefer edible treats over human tactile contact in an operant learning task [31]. Notably, the current exploratory study included only four horses per training method and additional studies are needed to further clarify the effect of training method and rewards on bonding and handling outcomes.

## 5. Conclusions

The results of the current pilot study could not demonstrate that horses develop an attachment bond after repeated interactions with their trainer, at least within the total duration of training in the current study (10 sessions of 15 min). However, the finding that horses were less aroused (heart rates were lower) during the post-tests may indicate either habituation to the test situation or a possible safe haven effect from a familiar person being present in the arena. Hence, until empirical evidence can demonstrate horse–human attachment, we should exploit the possibility that training outcomes are the result of affective state and attachment and not only of simple stimulus-response based interactions that influence trained responses.

We encourage further studies into the modalities that shape horse–human relationships and propose to address individual characteristics and variations in behavioural responses in both horses and humans under test conditions. Specifically, and in line with attachment theory, it would be important to account for the style of attachment because this determines which features of attachment may actually be expressed. Moreover, experimental designs should expose horses to different test situations (e.g., including human auditory cues to ensure recognition) and need to take into consideration the motivational systems of horses and their origin, their level of training (i.e., unhandled versus handled horses), training methods with information on reinforcement rates and previous experience with humans, including number of main caretakers.

## Figures and Tables

**Table 1:** Description of standardised exercises during training. Each task corresponds to one training session (total 10 sessions/horse spread over 10 days, lasting 15 min/session) and included a repetition of the previously learned task except for session 1.

Session	Task	Description
1	Park 1	The horse is trained to remain immobile when the trainer is stepping 2 m away from the horse while facing it (lead-rope is hanging loose).
2	Step backward	The horse is cued to take at least one step back on cue (lead pressure is applied in posterior direction).
3	Step forward 1	The horse is trained to start walking forward for at least three steps on cue (lead pressure is applied in anterior direction).
4	Park 2	The horse is trained to remain immobile when the trainer is stepping 2 m away from the horse while facing forward (lead-rope is hanging loose).
5	Move hindquarter	The horse is trained to offer a single step laterally with its hind leg on cue (whip tap on hock).
6	Step forward 2	The horse is trained to start walking at least a single step forward on cue (whip tap on croup).
7	Lower head	The horse is trained to lower its head on signal and maintain this position for at least 5 s (downward lead pressure).
8	Move forehand	The horse is trained to move laterally and cross the forelegs in a single step on cue (whip tap on shoulder).
9	Step forward 3	The horse is trained to start walking at least a single step forward on cue (whip tap on ribcage).
10	Turn head	The horse is trained to move its head to the side on cue and maintain the head position for 5 s (lead pressure sideways).

**Table 2:** Ethogram of behaviours in the fear and handling tests, carried out before (pre-test) and after (post-test) the training period. All behaviours were recorded in durations (s) as well as frequencies (resistance behaviour) and it was always noted whether the horse was closest to the stranger or the trainer.

Behaviour	Description
Fear test	
Location centre area ^1^	Time spent in the centre area. Started with at least two forelegs crossing the 2 m line and ended when the horse left the centre area, stepping outside the 2 m line with at least two forelegs.
Investigate object	Standing still within 1 m radius of the object with neck hold horizontally or lowered with the head and neck oriented toward the object, may include sniffing or touching the object.
Investigate person	Standing still within 1 m radius of the person with neck hold horizontally or lowered with the head and neck oriented toward the person, may include sniffing the person, with or without physical contact.
Handling test	
Resistance ^2^	Not standing still or not moving forward when requested, stopping, moving sideways, stepping backward, accelerating forward at the direction and speed cued by the handler (Stations A–E) or turning head in a 90-degree angle or tossing head upwards or downwards while handler uses lead pressure to cue the horse to back-up (Station D).

^1^ Centre area corresponds to the middle of the arena as indicated in Figure 2. ^2^ Recorded during approach (2 m distance) of each station and during and directly after encounter of the novel objects.

**Table 3:** Responses of horses in the pre- and post-tests (fear and handling), regardless of training method and in relation to stranger (S) and trainer (T). Values are presented as medians (range) or mean ± SD and correspond to durations (s), frequencies (f), and bpm (HR).

Tests	Variables	Pre-Test	Post-Test	*p*-Value
Fear	Location centre area S (s)	22.9 (2.5, 46.4)	9.0 (0, 27.0)	0.02
	Location centre area T (s)	23.0 (2.6, 56.7)	7.4 (0, 25.0)	0.17
	Investigate object S (s)	0.5 (0, 7.3)	0.2 (0, 6.1)	0.15
	Investigate object T (s)	3.3 (0, 10.5)	0.0 (0, 5.5)	0.83
	HR-mean (bpm)	85.6 ± 26.3	76.0 ± 25.9	0.03
	HR-max (bpm)	147.3 ± 22.5	122.7 ± 37.7	0.03
Handling S	Resistance behaviour (f)	2.5 (1.0, 4.8)	3.0 (1.3, 4.0)	>0.9
	Resistance behaviour (s)	6.7 (4.4, 15.3)	8.9 (2.3, 11.6)	0.72
	Time passing parkour (s)	96.8 (92.6, 103.6)	96.1 (93.7, 109.1)	0.41
	HR-max (bpm)	113.3 ± 33.9	77.3 ± 14.1	0.01
Handling T	Resistance behaviour (f)	3.0 (2.0, 4.8)	2.0 (1.0, 4.5)	0.26
	Resistance behaviour (s)	8.1 (4.5, 13.9)	4.6 (2.8, 10.8)	0.26
	Time passing parkour (s)	98.2 (94.5, 116.9)	97.9 (92.1, 104.9)	0.20
	HR-max (bpm)	98.6 ± 18.5	84.9 ± 21.4	0.02

**Table 4:** Behaviour and HR of horses during the fear and handling post-tests according to treatment (NR = negative reinforcement; NR + PRf = negative reinforcement plus positive reinforcement using food as rewards; NR + PRs = negative reinforcement plus positive reinforcement using wither scratching as rewards) and in relation to stranger (S) and trainer (T). Values are presented as medians (range) or mean ± SD and correspond to durations (s), frequencies (f), and bpm (HR).

Tests	Variables	NR	NR + PRf	NR + PRs	*p*-Value
Fear	Location centre area S (s)	2.1 (0, 14.4)	27.0 (6.2, 141.3)	6.9 (0, 24.3)	0.37
	Location centre area T (s)	7.9 (0, 36.5)	7.4 (0.6, 133.7)	11.4 (0, 25.0)	>0.9
	Investigate object S (s)	1.2 (0, 2.5)	4.3 (0, 9.9)	0.2 (0, 5.5)	>0.9
	Investigate object T (s)	0.0 (0, 4.1)	1.8 (0, 9.7)	2.8 (0, 7.6)	0.71
	HR-mean (bpm)	83.0 ± 32.2	75.5 ± 21.4	72.8 ± 26.8	0.86
	HR-max (bpm)	125.0 ± 36.0	134.8 ± 37.8	108.3 ± 39.2	0.62
Handling S	Resistance behaviour (f)	3.5 (0.8, 5.5)	2.5 (1.3, 3.0)	3.5 (1.5, 4.0)	0.22
	Resistance behaviour (s)	8.1 (1.3, 11.2)	7.0 (2.6, 9.6)	13.4 (3.2, 26.4)	0.37
	Time passing parkour (s)	96.1 (90.9, 101.9)	94.0 (93.3, 95.0)	112.8 (103.6, 153.3)	0.02
	HR-max (bpm)	77.5 ± 16.6	74.5 ± 16.3	83.3 ± 11.1	0.71
Handling T	Resistance behaviour (f)	2.5 (0.5, 3.0)	3.0 (0.3, 5.8)	2.0 (1.3, 7.3)	0.71
	Resistance behaviour (s)	4.7 (1.1, 8.9)	7.1 (0.8, 17.5)	5.2 (2.8, 38.4)	>0.9
	Time passing parkour (s)	92.4 (88.4, 100.0)	101.1 (91.3, 113.9)	100.4 (94.7, 146.7)	0.37
	HR-max (bpm)	96.3 ± 16.0	83.5 ± 23.2	76.0 ± 21.4	0.40

## Data Availability

Raw data supporting the findings of this pilot study are available from the corresponding author [E.H.] on request.
